# Manifestaciones orales de la viruela símica (MPX). Una revisión de literatura

**DOI:** 10.21142/2523-2754-1204-2024-221

**Published:** 2024-11-23

**Authors:** Harvy Yassbeck Cárdenas Machuca, Xiomara Nicole Marchena Gómez, Leyla Ruth Araucano Huaman, Melany Daniela Coanqui Astete, Jhon Paul Iakov Mezarina Mendoza

**Affiliations:** 1 Universidad Nacional Mayor de San Marcos, Facultad de Odontología. Lima, Perú. harvy.cardenas@unmsm.edu.pe , xiomara.marchena@unmsm.edu.pe , leyla.araucano@unmsm.edu.pe , melany.coanqui@unmsm.edu.pe , jmezarinam@unmsm.edu.pe Universidad Nacional Mayor de San Marcos Universidad Nacional Mayor de San Marcos Facultad de Odontología Lima Peru harvy.cardenas@unmsm.edu.pe xiomara.marchena@unmsm.edu.pe leyla.araucano@unmsm.edu.pe melany.coanqui@unmsm.edu.pe jmezarinam@unmsm.edu.pe

**Keywords:** viruela símica, viruela del simio, orthopoxvirus, monkeypox virus, manifestaciones bucales, odontología, monkeypox, monkey pox, orthopoxvirus, monkeypox virus, oral manifestation, dentistry

## Abstract

La viruela símica es una enfermedad viral que afecta a animales y humanos, similar a la viruela humana. Fue descubierta en 1958 en macacos en Dinamarca y en humanos en 1970, en el Congo, principalmente presente en las selvas de África central y occidental.

**Objetivo:**

Identificar las principales manifestaciones orales de la viruela símica mediante una revisión de la literatura.

**Materiales y métodos:**

Se realizó una investigación y recopilación de información bibliográfica especializadas en el tema, en buscadores científicos como PubMed, Medline, EBSCO, LILACS, Elsevier.

**Resultados:**

La revisión se realizó a base de 49 artículos encontrados que cumplieron los criterios de selección.

**Conclusión:**

La viruela del mono africana preocupa globalmente por casos en el hemisferio occidental. Síntomas en 2-4 semanas, principalmente en la cavidad oral: úlceras en labios y mucosa, que afectan la nutrición. Riesgo mayor en niños, adultos jóvenes y personas inmunodeficientes. Síntomas: fiebre, inflamación de ganglios y dolores musculares, en etapas pre-eruptiva y eruptiva.

## Abstract (en)

Simian smallpox is a viral disease affecting animals and humans, similar to human smallpox. It was discovered in 1958 in macaques in Denmark and in humans in 1970 in the Congo, mainly present in the jungles of central and western Africa.

**Objective:**

To identify the main oral manifestations of simian smallpox trough a systematic review.

**Materials and Methods:**

A research and compilation of specialized bibliographic information on the subject was carried out in scientific search engines such as PUBMED, MEDLINE, EBSCO, LILACS, ELSEVIER.

**Results:**

The review was carried out on the basis of 49 articles found that met the selection criteria.

**Conclusion:**

African monkeypox is of global concern for cases in the Western Hemisphere. Symptoms in 2-4 weeks, mainly in the oral cavity: ulcers in lips and mucosa, affecting nutrition. Increased risk in children, young adults and immunocompromised persons. Symptoms: fever, swollen glands and muscle aches, in pre-eruptive and eruptive stages.

## INTRODUCCIÓN

La viruela símica es una enfermedad infecciosa viral de ADN bicatenario, del género *Orthopoxvirus* de la familia Poxviridae, la cual puede transmitirse entre animales y seres humanos. Dentro del mismo género, se encuentra el virus de la variola (causante de la viruela humana), con el que tiene ciertas similitudes en las características clínicas. La enfermedad se describió por primera vez en 1958, cuando se presentó en macacos de laboratorio en Dinamarca; por ello se le denominó viruela de mono ^1^^-^^6^, y se reportó en humanos por primera vez en 1970, en un niño de 9 meses en la República del Congo ^7^^-^^9^. Desde ese momento, se han reportado casos de viruela símica dentro de África, específicamente en las selvas tropicales de África central y occidental, de donde se considera endémica ^10^.

La viruela del mono, al ser una enfermedad zoonótica, puede propagarse de animales a seres humanos por contacto directo con sangre, fluidos corporales y las lesiones mucocutáneas de animales infectados ^11^^,^^12^. Por otro lado, puede transmitirse entre seres humanos por contacto físico con personas infectadas o materiales contaminados, a través de gotitas respiratorias o contacto directo con el exudado de la lesión. Esta enfermedad se caracteriza por tener una presentación clínica similar a la viruela humana, se manifiestan a través de fiebre, linfadenopatía y erupciones cutáneas que tienen una duración de 2 a 4 semanas ^13^^,^^14^. Estas erupciones en la piel comienzan en forma de máculas, luego se convierten en pápulas y vesículas que eventualmente forman costras. Asimismo, el cuadro clínico implica dolores de cabeza, escalofríos, dolor de garganta, malestar, fatiga y dolor de espalda ^22^^,^^33^.

El primer suceso registrado fuera del contexto africano tuvo lugar en 2003 en los Estados Unidos y estuvo relacionado con la importación de una mascota exótica que provenía de Ghana ^2^^,^^3^. En los últimos años, se han registrado casos aislados y grupos pequeños de viruela de mono en el Reino Unido (2018 y 2019), Israel (2018), Singapur (2019) y Estados Unidos (2021). Todos estos incidentes tienen relación con viajes a Nigeria, siendo este país el que ha experimentado brotes nuevos de viruela de mono desde 2017 ^15^.

En mayo de 2022, en un hospital del Reino Unido, se registró a un paciente con antecedentes de haber viajado a Nigeria, el cual presentaba una erupción cutánea inexplicable, con las pruebas moleculares correspondientes que corroboraron el diagnóstico de viruela de mono. En los meses siguientes, se hallaron varios casos en diferentes países, con grupos significativos en Inglaterra, Alemania, Francia y Portugal. A diferencia de los anteriores brotes, no había un vínculo establecido entre las personas infectadas. Aunque todavía existe incertidumbre con respecto a la forma de transmisión y la infección, parece que el virus se propaga a través del contacto físico directo ^2^.

La Organización Mundial de la Salud (OMS) declaró el 20 de mayo de 2022 la alerta epidemiológica de casos de MPX para países no endémicos, a raíz de un brote que inició en Inglaterra y en los días posteriores, en forma creciente se describieron casos en España, Portugal, Alemania y EE. UU., lo que generó la alarma internacional de esta nueva enfermedad potencialmente infecciosa, obligando al mundo a realizar una vigilancia internacional ^13^. Hasta el momento, se han reportado más de 95 912 casos confirmados en 118 países ^16^. En el Perú, el primer caso de MPX fue reportado en junio de 2022 por el Centro Nacional de Epidemiología y desde esa fecha el aumento de casos ha sido considerable. Hasta la fecha se han reportado 3849 casos confirmados, de los cuales más del 70% están en el departamento de Lima, con 20 fallecidos ^17^^,^^18^. El objetivo de esta revisión de literatura fue documentar las principales manifestaciones orales más prevalentes en casos confirmados de MPX hasta el momento, y de esta manera advertir sobre las lesiones orales a profesionales odontólogos.

## MATERIALES Y MÉTODOS

**Figura 1:**
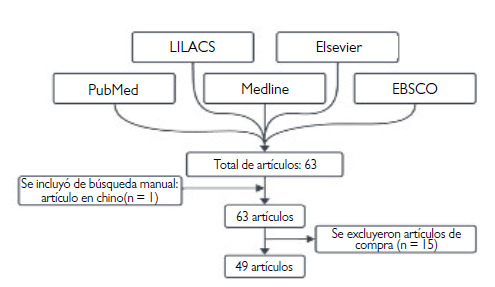
Diagrama de flujo de la selección de artículos.

En la presente investigación, se realizó una revisión de literatura científica que abarca desde septiembre de 2022 hasta marzo de 2024. Los documentos se consultaron en las siguientes bases de datos biomédicos: PubMed, Medline, EBSCO, LILACS y Elsevier. Se utilizaron las siguientes palabras clave: monkeypox, monkey pox, Orthopoxvirus, monkeypox virus, oral manifestation, odontología. Se completó con una búsqueda manual en libros y otras revistas no relacionadas con la odontología. Los criterios de inclusión para la presente revisión fueron artículos originales, reportes de casos clínicos, comunicaciones breves, cartas al editor, revisiones sistemáticas. Se buscó en idioma español, inglés y portugués, sin restricciones en los años de publicación. Los resultados de esta búsqueda dejaron un total de 63 artículos (español, inglés, portugués) y en la búsqueda manual se encontró 1 artículo en chino. Se excluyó 15 artículos debido a la imposibilidad de acceder a los estudios, por lo que al final la presente revisión incluyó 49 estudios (Figura 1).

## RESULTADOS

### Manifestaciones clínicas generales

La viruela símica en la mayoría de las personas se recupera en pocas semanas sin necesidad de algún tratamiento; sin embargo, la enfermedad puede ser más grave en niños, mujeres embarazadas y personas inmunodeprimidas ^19^.

La viruela símica presenta un período de incubación que puede variar entre 5 y 21 días. Para conocer las manifestaciones clínicas, es necesario comprender el curso de la enfermedad, el cual se divide según la presencia de aparición de las erupciones:

• Etapa preeruptiva. Los signos y síntomas duran entre 2 y 5 semanas. Se presentan fiebre y sarpullido entre 1 a 3 días antes de las erupciones cutáneas. El cuadro febril está acompañado de dolor de cabeza severo, dolor de espalda, mialgia, malestar general y postración; también se suele apreciar agrandamiento de los ganglios linfáticos ^10^^,^^29^. Además, el dolor en la boca puede ser el primer indicio de viruela del simio ^5^ (Tabla 1).

**Tabla 1:** Principales signos y síntomas de la viruela símica

Primera fase	Segunda fase
Fiebre Letargo Astenia Escalofríos Dolor de cabeza Inflamación de ganglios Mialgia Dolor de espalda Linfoadenopatías	- Evolución de las erupciones: mácula, pápulas, vesículas, pústulas y umbilicación; finalmente, costras y descamación - Tendencia centrífuga: inicia en el rostro y se dirige a las manos y pies; además, afectan a las mucosas orales, genitales, conjuntiva y córnea. - En algunos casos, las erupciones inician en los genitales - Celulitis, abscesos, necrosis de tejidos blandos - Neumonía severa - Infección corneal - Vómitos y diarreas - Septicemia - Encefalitis

• Etapa eruptiva. Inicia 5 días después de haberse presentado fiebre. La erupción cutánea aparece, generalmente, primero en la cara (95% de los casos), luego en las palmas de las manos y las plantas de los pies (en el 75% de los casos). También se ven afectadas las mucosas orales (en el 70% de los casos), los genitales (30%) y las conjuntivas (20%), así como la córnea ^10^^,^^19^^,^^30^ (Tabla 1).

Al igual que con la viruela, la lesiones se desarrollan más o menos simultáneamente, y evolucionan juntas a través de etapas de máculas, pápulas, vesículas y pústulas, antes de umbilicarse, secarse y descamarse. Sin embargo, estas pueden aparecer simultáneamente ^19^^,^^20^ (Tabla 1).

Las complicaciones más frecuentes van desde dificultad respiratoria, diarrea o vómitos hasta una encefalitis, infecciones en la córnea o bronconeumonía. La mortalidad en los brotes aumenta en niños, jóvenes y en personas inmunocomprometidas ^19^^,^^20^ (Tabla 1).

La viruela símica endémica de África se ha convertido en una preocupación mundial, por la aparición de casos esporádicos en regiones del hemisferio occidental ^19^. La transmisión del animal al humano se produce mediante arañazos o mordeduras de los animales infectados, o por el consumo de la carne de estos animales. Por otro lado, la transmisión entre personas es causada por fomites respiratorios contaminados o contacto directo con las lesiones de una persona infectada ^3^^,^^4^; sin embargo, aún no se ha podido verificar el trayecto de transmisión salival ^20^.

### Manifestaciones clínicas intraorales

Las manifestaciones orales que se encontraron en la revisión de la literatura se detallan a continuación: úlceras en boca, lesiones y úlceras en la mucosa oral. Las manifestaciones clínicas intraorales están basadas en los resultados que se obtuvieron al analizar la literatura; entre ellas tenemos las lesiones y úlceras que se presentan en la mucosa bucal, lo que aumenta las posibilidades de deshidratación entre los pacientes ^5^^,^^11^^,^^19^^,^^20^^,^^22^^,^^23^^,^^26^^-^^28^^,^^31^^,^^33^^,^^40^^,^^41^^,^^45^^,^^47^. Las lesiones orales precedieron al desarrollo de la erupción cutánea por uno o más días. Además, estas parecían tener una distribución algo medial a lo largo de la cara anterior de la lengua ^32^. Las úlceras se desarrollan en el labio, lengua, faringe, garganta y perioralmente, y van progresando desde una fase macular hasta una pustulosa ^5^^,^^19^^,^^20^^,^^27^^,^^40^^,^^41^^,^^47^. También hay presencia de linfadenopatía amigdalina y lingual que, por lo general, se presenta de forma temprana de 1 a 2 días después de hacer fiebre o la aparición de erupciones ^20^^,^^23^^,^^41^, producto de esta inflamación hay disfagia y dolor de boca y garganta ^11^^,^^20^^,^^37^^,^^38^^,^^40^^,^^47^. Por último, antes de que aparezca la erupción en la piel, una persona verá desarrollarse lesiones en la lengua y la boca; estas lesiones se llaman exantema y se presentan en boca y faringe ^44^; sarpullido en mucosa oral ^40^; lengua eritematosa ^43^; edema de úvula y paladar blando; edema petequial en paladar duro ^33^ y lesiones periorales ^19^^,^^20^ (Tabla 2).

**Tabla 2:** Manifestaciones clínicas intraorales

Lesiones intraorales	Autores
Lesiones y úlceras en la mucosa oral	- Kaler *et al*. - Franco *et al*. - Thornhill *et al*. - Gandhi *et al*. - Long *et al*. - Riad *et al*. - Huang *et al*. - Cardozo *et al*. - Damon - Alegre *et al*. - Benslama *et al*. - Rocha *et al*. - Vaughan *et al*. - Sookaromdee y Wiwanitkit
Úlceras en labio	- Franco *et al*. - Riad *et al*. - Damon - Benslama *et al*.
Linfoadenopatía lingual	- Franco *et al*. - Riad *et al*. - McCollum y DamonI - Benslama *et al*.
Enantema	- Long *et al*. - Damon
Lesiones ulcerosas en lengua	- Kaler *et al*. -Benslama *et al*. - Sookaromdee y Wiwanitkit
Disfagia	- McCollum y Damon - Issa *et al*. - Benslama *et al*.
Dolor de garganta	- Franco *et al*. - Gandhi *et al*. - Issa *et al*
Lesiones vesículo-ulcerativas	- Gandhi *et al*. - Joseph y Anil
Úlceras en garganta	- Long *et al*. - RECIAMUC
Sarpullido en mucosa oral	- Benslama *et al*.
Lesión pustulosa	- Rocha *et al*.
Úlceras en la faringe y agrandamiento amigdalino bilateral	- Erez *et al*.
Dolor de boca	- Sookaromdee y Wiwanitkit
Lengua eritematosa	- Gong *et al*.
Exantema	- Ogoina *et al*.
Edema de úvula/paladar blando, edema petequial en paladar duro y úlceras aftosas del paladar blando	- Alegre *et al*.
Amigdalitis	- Gandhi *et al*. - Rocha *et al*.
Lesiones periorales, faringitis y dolor de lengua	- Gandhi *et al*.

### Cuidados en la práctica dental

Los profesionales de la salud bucal corren un mayor riesgo, ya que se encuentran en contacto directo con lesiones de MPX o la exposición a gotas de saliva y aerosoles durante los procedimientos dentales ^35^. Frente a esta situación, ellos deben conocer y estar atentos a los signos y síntomas de la viruela del mono para poder evitar su contagio al momento de la atención ^48^.

La Agencia de Seguridad Sanitaria del Reino Unido nos brinda algunas recomendaciones como el uso de mascarillas N95 respiradores FFP3, vestimenta resistente a líquidos y protección para los ojos. Preferiblemente, el tratamiento dental debe suspenderse hasta que el paciente ya no sea diagnosticado con el virus; sin embargo, si el tratamiento es esencial en casos agudos, los pacientes deben ser tratados en una habitación aislada. Además, se debe realizar un lavado correcto de manos, así como la esterilización del instrumental usado ^49^.

## DISCUSIÓN

Yinka *et al*. ^21^ mencionan que las fuentes zoonóticas del brote actualmente se desconocen y no está claro qué cambios ambientales o ecológicos, si los hubiere, podrían haber facilitado su resurgimiento repentino. Peters *et al*. ^42^ señalan que las manifestaciones orales de la infección por viruela del mono se informan con poca frecuencia porque estos signos aparecen en una etapa temprana de la enfermedad. Sin embargo, este estudio ha permitido identificar y documentar las principales manifestaciones orales de la viruela símica, y proporciona una base valiosa para el diagnóstico temprano y el manejo clínico.

Varios autores mencionan que los signos más relevantes para el diagnóstico de la enfermedad son las llagas, úlceras, mialgia, linfoadenopatías y fiebre. No obstante, Sookaromdee y Wiwanitkit ^31^^,^^40^^,^^41^^,^^47^ indican que el único primer síntoma podría ser el dolor de boca, lo que sugiere que las manifestaciones orales serían cruciales para la detección precoz. Este hallazgo subraya la necesidad de que los profesionales de la salud bucal estén atentos a los signos orales iniciales para evitar complicaciones graves y mejorar los resultados del tratamiento.

Petersen *et al*. (2022) ^34^ mencionan que la mayoría de los datos actuales sobre la viruela del mono provienen de reportes de casos o brotes individuales, así como de la vigilancia pasiva intermitente, por lo que se deberían realizar más investigaciones. Además, señala que no existe evidencia de que la transmisión de persona a persona por sí sola pueda sustentar las infecciones zoonóticas en humanos. Este estudio contribuye a llenar ese vacío al proporcionar evidencia detallada sobre las manifestaciones orales y su impacto en los pacientes, lo que podría informar futuras investigaciones y políticas de salud pública.

Las manifestaciones orales identificadas, como las úlceras en los labios y la mucosa oral, tienen implicaciones significativas para la práctica odontológica. Los profesionales de la salud bucal deben estar equipados con el conocimiento necesario para reconocer estos signos tempranos y aplicar medidas preventivas efectivas. La Agencia de Seguridad Sanitaria del Reino Unido recomienda el uso de mascarillas N95, respiradores FFP3, vestimenta resistente a líquidos y protección para los ojos. Además, se debe suspender el tratamiento dental hasta que el paciente no sea diagnosticado con el virus, a menos que sea absolutamente necesario.

### Limitaciones

Aunque este estudio ha proporcionado información valiosa, existen limitaciones que deben ser consideradas. La dependencia de la literatura existente y la posible falta de acceso a ciertos estudios podrían haber limitado la comprensión completa de las manifestaciones orales de la viruela del mono. Futuras investigaciones deberían centrarse en estudios longitudinales y ensayos clínicos para evaluar intervenciones específicas en pacientes con viruela del mono, así como en la vigilancia activa para detectar y controlar futuros brotes.

Durante la elaboración de esta revisión, enfrentamos algunas limitaciones que podrían influir en la interpretación de los hallazgos. En primer lugar, la dependencia de la literatura existente, compuesta mayoritariamente por informes de casos y estudios descriptivos, limitó nuestra capacidad para extraer conclusiones más generales sobre las manifestaciones orales de la viruela símica. Por otro lado, la mayoría de los estudios incluidos provienen de ciertas regiones geográficas, lo que podría introducir un sesgo y dificultar la generalización de los resultados a otras poblaciones. Estas limitaciones subrayan la necesidad de realizar futuras investigaciones más robustas y diversificadas que permitan una mejor comprensión y manejo de las manifestaciones orales asociadas a la viruela símica.

## CONCLUSIONES

La viruela del mono de África se ha convertido en una preocupación mundial debido a la aparición de casos esporádicos en regiones del hemisferio occidental, con síntomas que suelen desarrollarse durante un período de 2 a 4 semanas. Esta revisión de literatura ha identificado que el 70% de los casos presentan manifestaciones clínicas en la cavidad oral, siendo las úlceras en los labios la principal manifestación oral, seguidas por las lesiones y úlceras en la mucosa oral, las cuales provocan disfagia y problemas de nutrición. El mayor riesgo lo tienen los niños, los adultos jóvenes y las personas inmunodeficientes. Los síntomas prevalentes se dividen en fase preeruptiva y frase eruptiva, e incluyen fiebre, linfadenopatías y mialgia. Las lesiones iniciales suelen aparecer en la mucosa oral antes de manifestarse en la piel, rostro y extremidades. Las lesiones ulcerosas en la boca, denominadas enantemas, son indicativas de la enfermedad y provocan dolor bucal, lo que puede ser un primer indicio de viruela del simio. Además, la transmisión de la viruela del mono incluye varias vías, desde arañazos y mordeduras de animales infectados hasta el contacto directo con lesiones mucocutáneas y fomites respiratorios contaminados. No obstante, aún existe incertidumbre con respecto al papel de la transmisión salival en la propagación de la enfermedad.

Esta revisión subraya la necesidad de que los profesionales de la salud bucal estén preparados para identificar y manejar las manifestaciones orales de la viruela del mono, aplicando medidas preventivas efectivas y manteniendo una vigilancia activa. Además, destaca la importancia de realizar investigaciones adicionales para comprender mejor la transmisión y evolución de la enfermedad, así como para desarrollar estrategias más efectivas de control y prevención. La vigilancia continua y la investigación activa son esenciales para prevenir futuros brotes y mejorar la respuesta global a la viruela del mono, a fin de garantizar una mejor salud pública a nivel mundial.

## Financiamiento:

Autofinanciado
